# Relationship between gut microbiota and host-metabolism: Emphasis on hormones related to reproductive function

**DOI:** 10.1016/j.aninu.2020.11.005

**Published:** 2021-01-04

**Authors:** Tarique Hussain, Ghulam Murtaza, Dildar H. Kalhoro, Muhammad S. Kalhoro, Elsayed Metwally, Muhammad I. Chughtai, Muhammad U. Mazhar, Shahzad A. Khan

**Affiliations:** a Animal Sciences Division, Nuclear Institute for Agriculture and Biology College, Pakistan Institute of Engineering and Applied Sciences (NIAB-C, PIEAS), Faisalabad, 38000, Pakistan; b Department of Animal Reproduction, Faculty of Animal Husbandry and Veterinary Sciences, Sindh Agriculture University, Tandojam, Sindh, 70050, Pakistan; c Department of Veterinary Microbiology, Faculty of Animal Husbandry and Veterinary Sciences, Sindh Agriculture University, Tandojam, Sindh, 70050, Pakistan; d Department of Animal Products Technology, Faculty of Animal Husbandry and Veterinary Sciences, Sindh Agriculture University, Tandojam, Sindh, 70050, Pakistan; e Department of Cytology & Histology, Faculty of Veterinary Medicine, Suez Canal University, Ismailia, 41522, Egypt; f Faculty of Animal Husbandry and Veterinary Sciences, University of Poonch, Rawalakot, 12350, Pakistan

**Keywords:** Gut microbiota, Host-metabolism, Steroid hormone, Reproductive function

## Abstract

It has been well recognized that interactions between the gut microbiota and host-metabolism have a proven effect on health. The gut lumen is known for harboring different bacterial communities. Microbial by-products and structural components, which are derived through the gut microbiota, generate a signaling response to maintain homeostasis. Gut microbiota is not only involved in metabolic disorders, but also participates in the regulation of reproductive hormonal function. Bacterial phyla, which are localized in the gut, allow for the metabolization of steroid hormones through the stimulation of different enzymes. Reproductive hormones such as progesterone, estrogen and testosterone play a pivotal role in the successful completion of reproductive events. Disruption in this mechanism may lead to reproductive disorders. Environmental bacteria can affect the metabolism, and degrade steroid hormones and their relevant compounds. This behavior of the bacteria can safely be implemented to eliminate steroidal compounds from a polluted environment. In this review, we summarize the metabolism of steroid hormones on the regulation of gut microbiota and vice-versa, and also examined the significant influence this process has on various events of reproductive function. Altogether, the evidence suggests that steroid hormones and gut microbiota exert a central role in the modification of host bacterial action and impact the reproductive efficiency of animals and humans.

## 1 Introduction

The interplay between gut microbiota and the host's physiology performs several functions that are comprised of growth, metabolism and immunity (Amato, 2016). The distinct gut microbiota composition is shaped by the environment and its host's genetic factors (Costello et al., 2009; Org et al., 2015). The human gut eco-system accommodates trillions of microbes that form a diverse community known as the gut microbiota. Evidence shows that the-interplay between gut microbiota and its host has proven health effects in Germ-free animals (Claus et al., 2008; Wikoff et al., 2009). The alteration of gut microbiota, through a change in diet or medication may influence the host's phenotype (Turnbaugh et al., 2006; Goodrich et al., 2014, 2016). The advanced approach of microbiome and metabolome could be a promising tool to determine the host-microbiome interactions (Turnbaugh and Gordon, 2008) and their outcomes. Evidence on metabolic approaches is complicated and limited studies have been conducted in this area on humans. The second approach uses 16S rRNA to confer the gut microbiota and its exceptional metabolic activation, and the fecal metabolome may help to better understand gut microbiota and its significance on health (Zierer et al., 2018). To expand our knowledge, it is better to apply the metagenomic shotgun sequencing (WMGS) approach which determines the taxonomic composition, function and its impact on the metabolic microbial community.

Cholesterol is the main deriver of sex steroid hormones in all animals. The male (testosterone) and female sex hormones (estrogen and progesterone), are chemically synthesized from the same pathway. The production of sex hormones relies on particular metabolizing enzymes which appear in a specific cell (Miller and Anchus, 2011). Hormone receptors are localized on the cell membrane, cytoplasm and nucleus. Once a hormone is bound with a specific receptor, it initiates a series of cellular responses; even a minute level of hormone exerts a positive impact (Molina et al., 2013). Estrogen is a vital hormone for female reproduction (Cook and Eidne, 1997; Liu et al., 2017), which controls different reproductive events, including estrus behavior, the enhancement of serum gonadotropin concentrations, ovulation, uterine propagation, and endometrial gland secretion (Nelson and Habibi, 2013; Hamilton et al., 2014). The estrogen delays osteopenia and is applicable in postmenopausal conditions subjected to osteoporosis. It also binds to a cognate receptor to exhibit its function in animals. Two categories of estrogen receptors (ER), alpha and beta, have been identified (Chauvigné et al., 2017). The third category, ERγ, has been documented in telo-fish (Paris et al., 2008). Androgen (testosterone) is a hormone responsible for male vigor and sexual integrity. During embryogenesis, it differentiates male characteristics from female (Murashima et al., 2015). Male hormones, with other counterparts, perform a variety of functions that enable a species to continue its reproductive life (Dutta et al., 2019). Androgen metabolism is involved in secretion, transport, tissue uptake, peripheral transformation, and excretion of C-19 steroids. The metabolic conversion of androgen produces steroids with different functions. The activity of androgen alters with the sex, age, thyroid status, weight and type of the tissue and is impacted by the metabolic process (Christopher, 2008). Progesterone is a key hormone that maintains pregnancy and regulates various functions in reproduction (Sawyer et al., 2019). The hypothalamus and pituitary glands are the main regulators of reproductive hormones (Dwyer, 2008). In female sheep and other animals, progesterone is synthesized by the corpus luteum, which exists over the surface of the ovary. Here, follicular waves develop into corpus luteum, which prepare the endometrium for embryo implantation (Field and Taylor, 2008). It can be used in early pregnancy if the endogenous progesterone level is deficient as to avoid early embryonic losses (Fernandez et al., 2018). This hormone is also utilized to induce cycling in anoestrus ewes. This treatment has proven successful in sheep for bringing herd uniformity and address infertility issues (Knights et al., 2011).

## 2 Nutrient regulation of gut microbiota

Nature has built up a strong relationship between gut microbiota and a host, which are mutually beneficial. Most gut microbiota behaves as commensals and is helpful for the host. Interestingly, gut microbiota does not remain the same; it alters due to the constant temperature and availability of substrates in the digestive tract. In an overactive metabolism, intestinal microbiota leaves unutilized nutrients for the host. In certain conditions, the relationship between the gut microbiota and it's host is disturbed (Woting et al., 2016).

Polyphenol compounds like flavonoids and others (Williamson, 2017) from different sources (Puupponen-Pimiä et al., 2002) possess strong antioxidant, anti-inflammatory and cancer prevention properties (Han et al., 2007). In vitro evidence suggests that polyphenols regulate gut microbiota (in humans) by suppressing pathogenic bacteria such as *Helicobacter pylori* and *Staphylococcus* spp., and assist beneficial bacteria (Selma et al., 2009). Previous literature highlights that polyphenols may alter gut microbes and microbial diversity (Etxeberria et al., 2015; Wang et al., 2018). Studies conclude that polyphenols possess prebiotic-like properties, which are the main cause of proven health effects in humans (Santino et al., 2017).

Vitamins are pivotal sources required in small amounts to maintain body homeostasis. They exert various functions and most importantly, serve as co-factors for enzymes. Vitamins cannot be synthesized by the body; they must be supplied through diet. However, a few are chemically synthesized by gut bacteria (Rowland et al., 2018). When a diet is lacking in vitamins, it causes health problems. Therefore, people may be advised to take high doses of specific vitamins to supplement dietary insufficiencies.

Minerals are required by the body in limited amounts and elicit active communication with gut microbiota (Skrypnik and Suliburska, 2018). Insufficient nutrients and abundances of minerals play a role in human diseases. Higher intakes of calcium alter the gut microbiota, which is connected with the lean phenotype (Zhang et al., 2019). In a human study, the consumption of 1,000 mg calcium/d for 8 weeks resulted in an increased *Clostridium* XVIII in fecal samples (Trautvetter et al., 2018). Other multigenerational studies revealed that maternal calcium enhances body weight gain in offspring (Garg et al., 2018). In a study of mice fed a high-fat diet, calcium supplemented at 5.25 g/kg elevated microbial diversity and bacterial spp. in the fecal microbiome of these animals (Aslam et al., 2016). High dietary calcium at 12 g/kg in a mother's diet was affiliated with reduced Verrucomicrobia in the offspring's gut. In contrast, insufficient calcium (2.5 g/kg) was linked with an increased Firmicutes-to-Bacteroidetes (F:B) ratio in the neonates (Li et al., 2018). Nutritional intervention for a short duration of 54 d, with a higher calcium supplementation was found to modulate the gut microbiota in the caecal samples in a high-fat diet mouse model. In this study, *Bifidobacterium* spp. exerted a negative impact due to plasma lipopolysaccharide levels, which showed a reduction in lipopolysaccharide production sites in the gut microbial pool (Chaplin et al., 2016).

Randomized and observational studies indicate that total high and saturated fat exerts an adverse effect on gut microbiota richness and diversity (Wolters et al., 2019). These results have been documented in rodent feeding trials with different fat concentrations at 44% and 72% enhanced F:B ratio of gut microbiota (Hamilton et al., 2015; Chen et al., 2018). The high-fat diet affects microbial abundance, the F:B ratio, and overall density. Further details of the nutrient absorption, their interaction with gut microbiota, and their clinical aspects are well-reviewed by Yang et al. (2020).

## 3 Bacterial metabolism of sex steroid hormones

Steroid hormones such as progesterone, estradiol, and testosterone regulate various reproductive events, such as apoptosis, inflammation and metabolism (Edwards, 2005). They are produced by gonads, adrenal glands and placenta thereafter, and act to target tissues to influence their activity via the blood stream (Wilson et al., 1998). These exert their function through specific or membrane receptors that activate signaling pathways (Scarpin et al., 2009; Cabrera-Muñoz et al., 2012). Steroids are conserved signaling molecules that regulate different physiological functions in animals like reproduction, mineral, lipid and glucose homeostasis. Errors in steroid hormone signals have been recognized as an inducer of several diseases (Masuzaki et al., 2001). Due to the conserved nature of steroids, they regulate several transcriptional genes (Beato et al., 2000). Notably, the balance between steroids and non-steroids is strongly mediated by hydroxysteroid dehydrogenases (HSD) (Blum, 2003). The prominent bacterial phyla which produce HSD enzymes are Actinobacteria, Proteobacteria, and Firmicutes. Large quantities of HSD producing bacteria are responsible for organizing gastrointestinal flora and others are derived from seawater, soil, and marine sediments (Kisiela et al., 2012).

The metabolomics approach unveiled that mice treated with streptomycin experienced an impaired intestinal homeostasis which ultimately influenced the intestinal metabolome. Approximately 87% metabolites decreased, including steroids, showing a relationship between intestinal microbiota and steroid metabolism (Antunes et al., 2011). An association has been made between steroid enzyme 21-dehydroxylation or 16α-dehydroxylation with intestinal microbiota but such a relationship is not reported in mammalian organisms (Bokkenheuser and Winter, 1980). The 17β suppression of androgen-regulated human intestinal microbiota plays an essential role in controlling testosterone and androgen concentration (Donova et al., 2005). It is worth noting that steroid metabolism is not only regulated by pathogens or bacteria in mammals and animals, but also by environmental bacteria (Göhler et al., 2008). Intriguingly, an enzyme 3α-HSD/carbonyl reductase (CR) is responsible for regulation of steroid metabolism (Kisiela et al., 2012).

The testosterone catabolic pathway is different from *Comamonas testosteroni* which has shown affiliation with *Steroidobacter denitrificans* (Leu et al., 2011). Bacteria released from sludge possess the capability to metabolize estradiol and testosterone hormones (Fahrbach et al., 2008). The environment is a source of natural and synthetic steroids, which may threaten the health status of humans and animals, influencing puberty and reproduction. Some bacterial species may degrade steroids, which might be helpful for bio-remediation of steroid-contaminated environments (Kisiela et al., 2012). Further, these bacteria could be used to remove pollution from the environment (Iasur-Kruh et al., 2011). Estradiol and estrone pass through livestock and other wastes. Most bacteria, once they degrade, produce estradiol and are transformed into estradiol then into estrone, which cannot degrade further (Jiang et al., 2010). The majority of these strains are unable to degrade further (Yu et al., 2007). The key enzymes in biodegradation may uncover microbial estrogen degradation pathways, which could be used as biomarkers of estrogen degradation taking place through microbes (Li et al., 2012). Such bacteria may formulate a mixture of different bacteria capable of destroying distinct classes of steroids and their associated components (Li et al., 2012).

## 4 Microbial regulation of reproductive hormones

Predictably, several processes are altered due to reproduction and variation in the female gut microbiome, throughout pregnancy. Alteration in gut microbial diversity and composition during pregnancy and lactation have been described in invertebrates (Trevelline et al., 2019; Elderman et al., 2018) including humans (Jost et al., 2014; Koren et al., 2012) and non-human primates (Mallot et al., 2018). Less information exists on the shifts of the gut microbiome during the entire reproductive state. Diet, behavior and other factors direct reproductive hormones, which are likely to bring these changes (Jost et al., 2014; Mallot et al., 2018). Steroid hormones such as progestagens and estrogens exert desired effects in pregnancy consisting of alteration in the inflammatory response, changes in maternal metabolism and cardiovascular function (Magness, 1998). Therefore, these hormones are the mediators of the gut microbiome function. Little evidence showed that estrogen also regulates gut microbial composition (Vieira et al., 2017). Some studies have determined that reproductive hormones and gut microbial community composition are impacted in cycling, pregnant, and lactating individuals (Antwis et al., 2019).

**Fig. 1:**
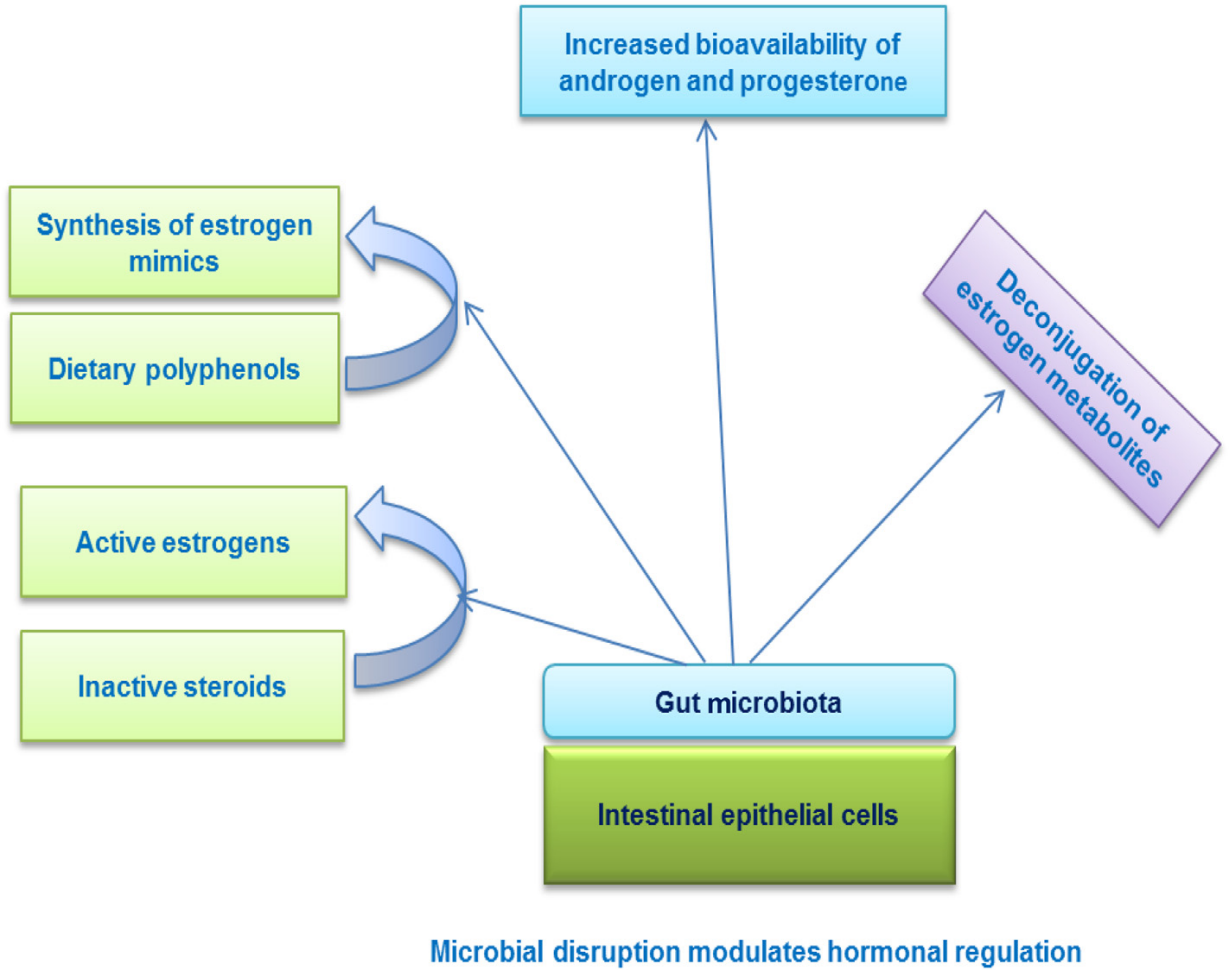
Alteration in gut microbiota mediates hormonal regulation.

Female reproduction is a phenomenon where more energy is utilized for reproductive and non-reproductive functions like behavioral and physiological processes (Ricklefs et al., 2010). Therefore, alteration in the gut microbiome during pregnancy and lactation may have the same job; commensal association with gut microbiota can accommodate females for energetic shortfalls during reproduction. But it is not clear how gut microbiota is involved in reproduction, nor the changes in the energy requirements of the host well-defined. Further, only one paper supports the evidence of gut microbiota contribution during the third trimester of pregnancy, showing evidence of an obesity-linked gut microbiome suggesting that the gut microbiome may enhance energy harvest but not contribute to the efficiency of energy uptake (Koren et al., 2012). On the other hand, microbial alteration may arise from other host or physiological deviations during gestation and lactation, therefore variations in gut microbiota may not have a direct relationship with energy metabolism (Amato et al., 2014). More evidence is required to address this issue. A study on wild Phayre's leaf monkeys revealed that the reproductive stage was influenced by the gut microbiome composition, which is driven by reproductive hormones. In females, reduced microbial diversity during pregnancy, along with fecal progesterone levels, were negatively linked with diversity. Dysbiosis in gut microbiota function influenced hormonal activity (Fig. 1). The season and phytoestrogen also influences gut microbiota indices. Overall, it indicated that progestagens, are responsible for shifting the gut microbiome in pregnancy and lactation (Mallott et al., 2020).

## 5 Microbial degradation of sex steroid hormones

There are over 1,000 varieties of steroids that have been reported in nature (Valitova et al., 2016; Zubair et al., 2016; Stonik and Stonik, 2018) comprising sterols (cholesterol, phytosterols and ergosterol), steroid hormones (17β-oestradiol, progesterone and testosterone) and bile acids (cholic acid). Cholesterol is the deriver of steroid hormones in animals. It consists of sex steroids hormones, glucocorticoids and mineralocorticoids. The chemical synthesis of steroids comprises the exclusion of cholesterol side chains and hydroxylation of the steroid nucleus chain (Ghayee and Auchus, 2007). The hydroxylation reactions need NADPH and molecular oxygen; thereby steroid biosynthesis takes places in an aerobic environment. Amid steroid hormones, like progesterone, it prepares a suitable environment for implantation and maintaining pregnancy. The conversion of progesterone into androgens comprises hydroxylation at C-17 and cleavage of side chains. Androgen is the male dominant hormone mostly produced in males as testosterone, dihydrotestosterone (DHT) and androstenedione (O'Connor et al., 2011). Estrogens are the regulators of the reproductive system and other secondary sex characteristics. Estrogen biosynthesis occurs by androgens through the loss of C-19 angular methyl group and the formation of an aromatic A-ring. The aromatization peruses 3 consecutive steps (Miyairi and Fishman, 1985). Aromatase such as P450 or CYP19 catalyses sequential hydroxylations of a C19 substrate via 3 molecules of each NADPH and molecular oxygen to yield one molecule of oestrogen (Praporski et al., 2009).

Steroids are present in plentiful sources in the environment and are crucial for microorganisms. Specific microorganisms such as bacteria (Donova and Egorova, 2012), can transform steroids, however; the ability to mineralize steroids is only limited to bacteria (Holert et al., 2018). The current focus of research has been concentrated on a variety of steroid degraders and their mechanisms. A culture-dependent approach is applicable for isolating steroid degrading bacteria from a different source and degraders such as Actinobacteria and Proteobacteria (Bergstrand et al., 2016). The steroid interactive mechanisms can be classified into 4 themes: 1) Use microbes to exclude sex hormones from polluted environments; 2) Microbial transformation of steroids for production of valuable steroid drug-using biotechnological approaches; 3) Steroid degradation is essential for some virulent bacteria; 4) Steroids regulate interplay amid bacteria and eukaryotic hosts (Vom Steeg and Klein, 2017). The detailed description of microbial degradation in aerobic, and an-aerobic environments, and involvement of particular pathways are well-reviewed by Chiang et al. (2019). Of note, bio-degradation products can be more detrimental to the parent compound (International Union of Pure and Applied Chemistry, 1993).

## 6 Gut microbiota, hormones and reproductive function

The first evidence of sex hormone involvement in gut microbiota was documented in the 1980s. The *Prevotella intermedius* uptake estradiol and progesterone that increase its development (Kornman and Loesche 1982). The altered response of ERβ, impairs intestinal microbiota (Menon et al., 2013). This phenomenon works on both sides, while several bacteria are employed in regulating steroid activity or alteration (Ridlon et al., 2013). The *Clostridium scindens* transforms glucocorticoids to androgens (male hormone) (Ridlon et al., 2013). The intestinal microbiota displays a prominent function in estrogen metabolism, while the application of antibiotics reduces estrogen levels (Adlercreutz et al., 1984). Moreover, a strong relationship has been observed in urinary estrogen and faecal microbiome abundance, and *Clostridia*, thus showing non-Clostridiales, and 3 genera within the Ruminococcaceae.

Mittelstrass et al. (2011) proposed that interaction between the endocrine system, gut bacteria and metabolism were dependent on sex differences in the fatty acid profile. Pregnancy is a period where gut microbiota is altered and cause several changes in host-hormonal concentration. Physiologically, estrogen, progesterone and leptin are greatly increased during pregnancy, whereas, adiponectin and pituitary gonadotropins are reduced (Newbern and Freemark, 2011). The gut microbiota composition causes disruption, particularly in the third trimester of pregnancy, and these changes alter the metabolism (Koren et al., 2012). As the pregnancy progresses, the positive impact of Proteobacteria and Actinobacteria is increased. During the last trimester of pregnancy, lower levels of inflammation are found and insulin function is more strongly influenced, compared to the first trimester. This experiment was conducted in germ-free mice where it showed that third phase pregnancy microbiota enhanced weight and had a peak inflammatory response compared with the first phase of pregnancy (Koren et al., 2012). It is presumed that these alterations might be linked with hormonal fluctuation. Impairment in gut microbiota may predispose a subject to disease associated with hormonal changes, such as polycystic ovary syndrome. Considering the theoretical perspective, poor diet may cause an imbalance in gut bacterial communities, enhance gut mucosal permeability and ultimately turn into stimulation of the immune system. After that, it increases serum insulin concentration, androgen levels and influences normal follicular development (Tremellen and Pearce 2012). A diet poor in carbohydrate has also been verified to mitigate the symptoms of the syndrome.

## 7 Contribution of gut microbiota in regulation of hormones

Gut microbiota exert their function as an endocrine since they may produce and metabolize several compounds that resemble hormones (Clarke et al., 2014). Gut microbiota can regulate sex hormones along with metabolites (Adlercreutz et al., 1979). Sex or gender variances are important factors elucidating the host-physiology and behavior features, but further research is needed on whether sex hormones are affiliated with intestinal microbes. Differences in the composition of commensal microbiota of male mice were observed when compared to females at the stage of puberty. Additionally, Markle et al. (2013) observed that circulating testosterone concentration enhancements was followed by commensal microbiota in female mice. Further, evidence unveiled the prominent influence of gonadectomy and hormone replacement on the composition of microbiota in mice (Org et al., 2016). These indications show that sex hormones have a significant impact on the gut microbiota of the host and systemic concentrations of estrogens. It has been observed that gut microbiota in women was distinct, and elevated ratios of hydroxylated estrogen metabolites to estradiol were found in their urine (Fuhrman et al., 2014).

Evidence suggests the association between the concentration of urinary estrogens and abundances and diversity of the fecal microbiome. Flores et al. (2012) highlighted that systemic estrogens showed a potent link with fecal Clostridia taxa and specific genera in the Ruminococcaceae family. It has been predicted that conjugated estrogens are eliminated through the urine or with bile in feces. Conjugated estrogens may be converted into specific bacterial genera, which reside in the human intestine, and possesses active beta-glucuronidase, which enhances the reabsorption of estrogen into the blood and reduces the removal from the body (Kwa et al., 2016). Hence, systemic amounts of estrogens and their metabolites may be impaired by particular genera of the gut microbiome. While few studies have reported evidence of connections of hormones with gut microbes, the connection of this has been implied in animal models (Menon et al., 2013). Further, limited studies have been conducted in humans to understand the alteration in bacterial communities due to sex hormones (Flores et al., 2012; Kwa et al., 2016). However, controversial results have been found in a few murine models (Wallace et al., 2018). Furthermore, the literature has placed an emphasis on estrogen (Flores et al., 2012; Menon et al., 2013; Lindheim et al., 2017) while only some studies documented the link between intestinal microbial composition and the concentration of testosterone (Al-Asmakh et al., 2014).

## 8 Gut microbiota regulates androgen metabolism in intestines

Testosterone is a hormone present in the systemic circulation of males, but is also found in females. The production of this hormone is regulated by gonadotropins such as luteinizing hormone (LH) and follicle-stimulating hormone (FSH), which is governed by a negative feedback of steroids (Anderson et al., 2018; Bélanger et al., 2003). Adrenal derived androgen, a precursor of dehydroepiandrosterone (DHEA), takes part in the synthesis of androgen in humans (Bélanger et al., 1989) though in mice it is not released. Testosterone in target tissues and liver may be further metabolized via phase-I reactions and phase-II reactions (Schiffer et al., 2018). The glucuronidated androgens are eliminated from the body in urine or by bile to the small intestine (Bélanger et al., 2003).

An in vitro study revealed that particular strains of bacteria were able to metabolize androgens, for example, transforming testosterone into DHT (Soory et al., 1995). The biological importance of gut microbiota in androgen metabolism and the amount of glucuronidated and free androgen is unidentified in distinct parts of the intestine. Current evidence shows the associations between gut microbiota and androgen levels in mice, while female mice received male intestinal contents, showing enhanced serum concentration. In this study, local androgen amounts were not identified in intestinal contents (Markle et al., 2013). The positive impact of androgen is well-described in androgen targeted tissues and the intestinal tract. A study was conducted to determine the evidence of unconjugated (free) and glucuronidated androgen concentration in contents of the small intestine having low bacterial density, and in the cecum and colon with increased bacterial density. Results revealed that gut microbiota contributed to intestinal metabolism and deglucuronidation of DHT and testosterone showed an increased level of strong androgen in colonic contents of young and normal men. Hormones and their associated relationship with gut microbiota are displayed in Table 1.

**Table 1:** Hormones and their known relationship with gut microbiota.

Functional classes	Hormones	Model	Outcome	Bacterial species	References
Bacterial growth and expressions	Estriol, estradiol	Quorum sensing	↓ Hormones bacteria virulence	*Agrobacterium tumefaciens* and *Pseudomonas aeruginosa*	Roshchina et al. (2010)
Bacterial growth and expressions	Estrogen, progesterone	Bacterial growth	Estradiol and progesterone promoted bacterial growth	*Platysaurus intermedius*	Kornman and Loesche (1982)
Host behavior	Corticosterone, adrenocorticosterone	Probiotics in rats and humans	Bacteria decreased stress hormones	*Lactobacillus helveticus* and *Bifidobacterium longum*	Messaoudi et al. (2011)
Host appetite and metabolisms	Ghrelin	Male rats	Specific bacteria significantly correlated with ghrelin	*Bacteroides* and *Prevotella* spp.	Queipo-Ortuno et al. (2013)
Host appetite and metabolisms	Ghrelin	Male rats	Specific bacteria negatively correlated with ghrelin	*Bifidobacterium, Lactobacillus* and *Blautia coccoides–Eubacterium rectale* group	Queipo-Ortuno et al. (2013)
Host appetite and metabolisms	Ghrelin	Prebiotics (oligofructose) in obese humans	Prebiotics decreased secretion of ghrelin	Promoted growth of *Bifidobacterium* and *Lactobacillus*	Parnell and Reimer (2009)
Steroid hormones and reproduction	Estrogen	Antibiotics	Antibiotics decreased estrogen levels		Adlercreutz et al. (1984)
Steroid hormones and reproduction	Estrogen	Humans	Correlations between urinary estrogen levels and fecal microbiome composition and richness	*Clostridia* taxa, including non-Clostridiales and 3 genera in the Ruminococcaceae family	Adlercreutz et al. (1984)
Steroid hormones and reproduction	Androgens	Enzymatic and kinetic studies	Bacteria converted glucocorticoids to androgens	*Clostridium scindens*	Winter et al. (1984)
Steroid hormones and reproduction	Testosterone	non-obese diabetic (NOD) mice	Microbes increase testosterone levels		Markle et al. (2013)

Table courtesy of Neuman et al. (2015).

## 9 Gut microbiota and progesterone significance in late pregnancy

Pregnancy is a period during which gut microbiota alteration takes place (Aagaard et al., 2012; DiGiulio et al., 2015). Some of these changes linked with gut microbiota are similar to changes that occur in metabolic disorders (Pitlik and Koren, 2017). The alterations consist of the enhanced richness of Proteobacteria, Actinobacteria, opportunistic pathogens, reduced bacterial derived short-chain fatty acid and in species abundances, with this whole scenario being pertinent as the pregnancy progresses (Koren et al., 2012). Progesterone is a dominant hormone of pregnancy that performs a central role in the maintenance of gestation (Mesiano, 2001). A study on the microbial shift of gut microbiota and its composition in women and mice during late pregnancy revealed that progesterone is also a dominant hormone that influences the microbial community. Progesterone abundant bacterial species such as *Bifidobacterium* have been reported to have a direct effect in vitro. Results concluded that progesterone enhanced *Bifidobacterium* richness in late pregnancy and can be used as a model (Nuriel-Ohayon et al., 2019).

## 10 Interplay between gut microbiota and estrogen hormone

**Fig. 2:**
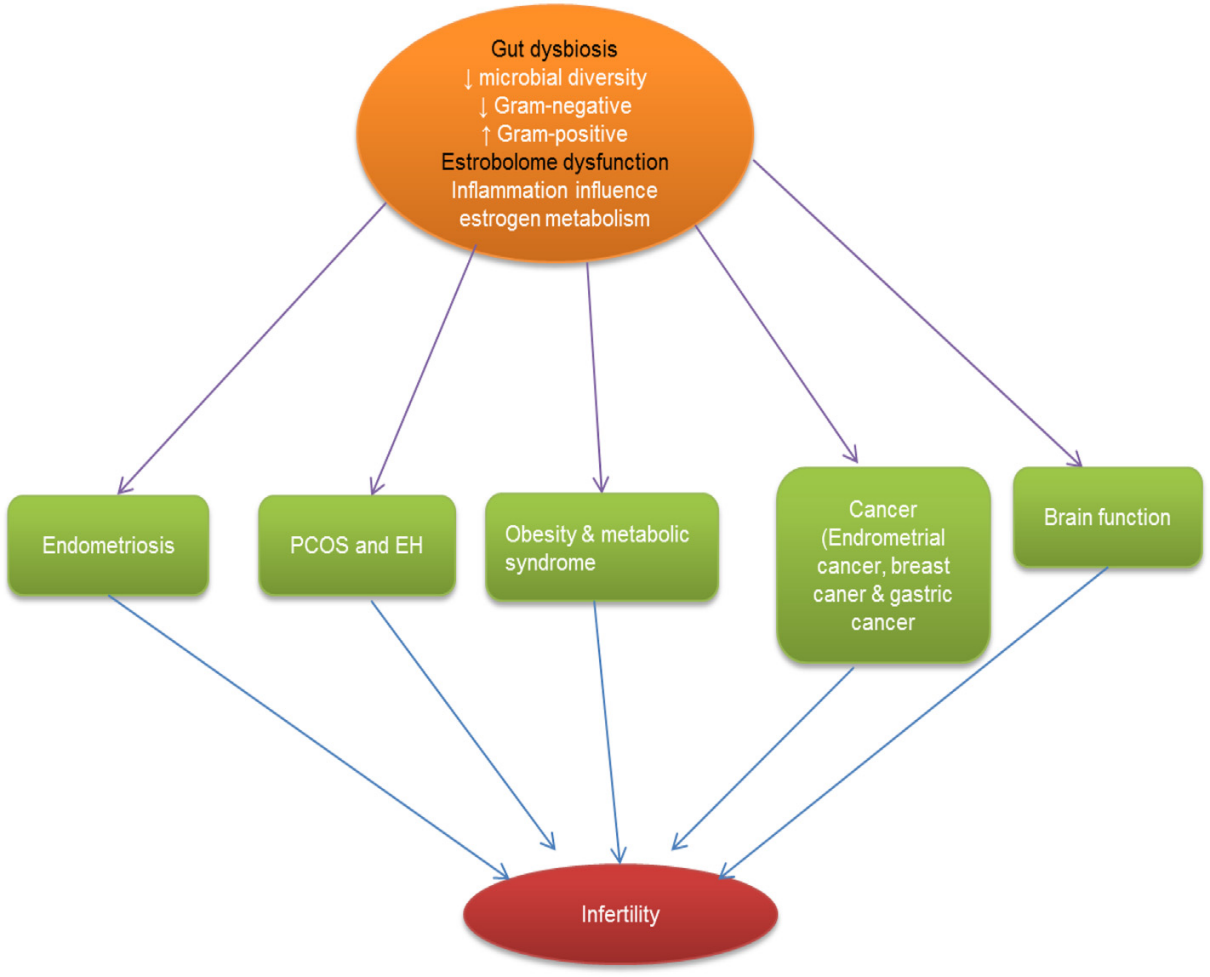
Relationship between estrogen and gut microbiome exerting physiological and clinical implications. PCOS = polycystic ovary syndrome; EH = endometrial hyperplasia.

The crosstalk between estrogens and microbiota display a key role in obesity, independent of each other. However, evidence indicates that estrogen and microbiota may jointly regulate weight gain and lipid deposition. Microbiota enables the metabolization of estrogen-like compounds (biologically active) and can enhance proliferation and growth of specific bacteria (Frankenfeld et al., 2014). For instance, daidzein, an isoflavonoid metabolized by gut bacteria to O-desmethylangolensin (ODMA) and S-equol (Frankenfeld et al., 2014) that seems structurally similar to estrogen, may trigger the stimulation of ERα or ERβ (Nakatsu et al., 2014 Usui et al., 2013). In addition, genistein, and glycitin can positively change the structure and composition of the fecal bacterial community in postmenopausal women, enhancing the level of beneficial Gram-positive *Bifidobacterium*, while suppressing Clostridiaceae, which could be the cause of many human diseases (Frankenfeld et al., 2014). Diets rich in phytoestrogens are known to have increased weight gain, and enhanced gut microbial metabolites of the estrogenic plant (lignans) in the blood have been linked to reducing weight rate and obese (Usui et al., 2013). Individuals which harbour bacteria may produce ODMA, and they are to be obese than those who are producers (Frankenfeld et al., 2014). More than 50% of obese subjects are equol non-producers (Nakatsu et al., 2014). The equol may help in glycemic control and lower low-density lipoprotein levels, representing a prominent role of bacteria in the stimulation of soy phytoestrogens, with respect to obesity (Usui et al., 2013). Hence, the role of phytoestrogens may be to limit the metabolic syndrome. Current literature has documented that supplementation of chalconoid isoliquiritigenin increases ovariectomy-related weight gain and metabolic syndrome in mice, irrespective of the activation of reproductive tissues (Madak-Erdogan et al., 2015). However, it is not clear whether the supplementation approach of isoliquiritigenin or lower affinity estrogens would modify gut microbiota and blood metabolites. Currently, intestinal microbiota genes allow for production of estrogen-metabolizing enzymes. These genes or the estrobolome can be used as a biomarker of many diseases (Kwa et al., 2016). Absorption of estrogen takes place through the liver by first phase-metabolism and bio-transformation via methylation, hydroxylation, and conjugation into metabolites of different ER. The estrobolome could modify transformed estrogens. For instance, a few bacterial spp. have been identified with β-glucuronidase activity, which can enhance intestinal reabsorption of estrogens. Elevated estrogen metabolites seem to be potentially linked with microbial diversity, in response to parent estrogens that exist in fecal stools (Flores et al., 2012), and enhanced parent estrogen levels in response to estrogen metabolites have been linked to a higher risk of breast cancer (Flores et al., 2012). The mutual interaction of estrogen with gut microbiota is displayed in (Fig. 2). The estrogen metabolite to parent estrogen ratio is an essential tool to determine obesity and metabolic diseases. The data conclude that gut microbiota is a robust biomarker for analyzing and suppressing estrogen-related problems.

## 11 Contribution of gut microbiota in breeding success, hormonal metabolites and on ovarian cyclic phase

Microbial communities in the host impart a vital function in whole organisms (Fitzpatrick et al., 2018; Hanning et al., 2015). Previous studies have documented that the microbiome regulates hormones and steroid function in human and model organisms. The microbiome performs different functions, including host hormones, and its function is also hampered by oestrogens, testosterone etc., which influence host gene expression (Evans et al., 2013; Mayer, 2011). Gut microbiota may act as an endocrine function, and it is responsible for the degradation of hormones, deviation of host gene expression, and may eventually influence the reproductive outcome (Asano et al., 2012; Kunc et al., 2016). Moreover, gut microbiota and adrenal hormones both are involved in reproduction, whereas the host-microbiome is also capable of influencing hosts fitness. The human gut microbiome regulates estrogen production via β-glucuronidase enzymes (Flores et al., 2012). Further, glucocorticoids may be transformed into androgens by specific human gut microbiome (Ridlon et al., 2013). An altered gut microbiome proceeds changes in estrogen levels, and the modification may change estrogen levels and consequently leading to infertility (Baker et al., 2017). The microbiome of the reproductive tract may influence pregnancy outcomes in humans, and further research is needed to determine how the microbiome impact a successful increase in the full term of pregnancies (Franasiak, 2015a, Franasiak and Scott, 2015b). A study by Miller et al. (2017), revealed a positive alteration in the vaginal microbiome of wild baboons (*Papio cynocephalus*) in gestation, cyclic and postpartum amenorrhea conditions. The researchers found that the microbiome changed throughout ovarian cycle, into a specifically different microbiome classified with an increased richness of *Streptococcus*, *Trichococcus*, *Sneathia* and *Bifidobacterium* during the ovulatory phase (Miller et al., 2017) although the relationship of these changes to reproductive success remains elusive.

A study by Antwis et al. (2019) demonstrated a significant relationship in black rhino gut microbiome composition observed in breeding success and ovarian cyclicity. Findings further indicated an altered microbiota level in gestation and lactation. Here, a significant association was found between gut microbiota and progestagen or glucocorticoid levels. Four genera, Aerococcaceae, Atopostipes, Carnobacteriaceae and Solobacterium, were investigated. These had a positive relation with breeding success during gestation and or lactation along with increased fecal progestagen metabolite concentrations.

## 12 Conclusion

Gut microbiota exerts a vital role in the maintenance of host physiology. They are involved in regulating steroid hormones and vice-versa. Bacteria have evolved a mechanism to utilize sex hormones and are implicated for degradation or chemical modification. This mechanism can be helpful by eradicating steroid hormones from a polluted environment. Deeper understanding is needed for particular enzymes and their mechanism for selection of specific bacteria for bio-remediation programs. The role of steroid hormones in reproduction is well understood but their interaction with gut microbiota is not completely explored. Here, we have presented a collection of evidence of the involvement of gut microbiota in the reproductive function. Gut microbiome in black rhino during pregnancy and post-parturition was modified, and about one third of bacteria genera presented a 10% correlation with progestagen or glucocorticoid concentration. This suggests that the probiotic approach may be helpful for increasing breeding success (Antwis et al., 2019). Further studies are warranted to explore how dietary intervention strategies can impact microbial augmentation.

Gut microbiome research is attracting huge attention. Hormones regulated by microbiota exhibit a positive impact on host behavior, metabolism and reproduction. As our knowledge deepens regarding the microbiome, we hope that more mechanisms will be identified regarding hormones consisting of novel interactions. Particular classes of bacteria are involved in mediating hormonal levels. These novel approaches in the future may become a great reality. To achieve this scenario, there is a need to verify the underlying mechanism, which defines bacterial strains and endocrine–microbiome interactions.

## Author contributions

T. Hussain, conceptualization; project administration; supervision; validation; visualization; roles/writing – original draft; writing – review & editing; G. Murtaza, D. H. Kalhoro, M. S. Kalhoro, E. Metwally, M. I. Chughtai, M. U. Mazhar, and S. A. Khan: validation; visualization, editing of the manuscript.

## Conflict of interest

We declare that we have no financial and personal relationships with other people or organizations that can inappropriately influence our work, there is no professional or other personal interest of any nature or kind in any product, service and/or company that could be construed as influencing the content of this paper..
